# Molecular Phylogeny of Endophytic Fungi from Rattan (*Calamus castaneus* Griff.) Spines and Their Antagonistic Activities against Plant Pathogenic Fungi

**DOI:** 10.3390/jof7040301

**Published:** 2021-04-15

**Authors:** Nurul Farizah Azuddin, Masratul Hawa Mohd, Nik Fadzly N. Rosely, Asyraf Mansor, Latiffah Zakaria

**Affiliations:** School of Biological Sciences, Universiti Sains Malaysia, Penang USM 11800, Malaysia; farizah0610@gmail.com (N.F.A.); masratulhawa@usm.my (M.H.M.); nfadzly@usm.my (N.F.N.R.); asyrafm@usm.my (A.M.)

**Keywords:** endophyte, spines rattan palm, phylogeny, biocontrol, plant pathogenic fungi

## Abstract

*Calamus castaneus* is a common rattan palm species in the tropical forests of Peninsular Malaysia and is noticeable by the yellow-based spines that cover the stems. This study aimed to determine the prevalence of fungal endophytes within *C. castaneus* spines and whether they inhibit the growth of fungal pathogens. Twenty-one genera with 40 species of fungal endophytes were isolated and identified from rattan palm spines. Based on molecular identification, the most common isolates recovered from the spines were *Colletotrichum* (*n =* 19) and *Diaporthe* spp. (*n =* 18), followed by *Phyllosticta* spp., *Xylaria* sp., *Trichoderma* spp., *Helminthosporium* spp., *Penicillium* spp., *Fusarium* spp., *Neopestalotiopsis* spp., *Arthrinium* sp., *Cyphellophora* sp., *Cladosporium* spp., *Curvularia* sp., *Bionectria* sp., and *Acremonium* spp. Non-sporulating fungi were also identified, namely *Nemania primolutea*, *Pidoplitchkoviella terricola*, *Muyocopron laterale*, *Acrocalymma fici*, *Acrocalymma medicaginis*, and *Endomelanconiopsis endophytica*. The isolation of these endophytes showed that the spines harbor endophytic fungi. Most of the fungal endophytes inhibited the growth of several plant pathogenic fungi, with 68% of the interactions resulting in mutual inhibition, producing a clear inhibition zone of <2 mm. Our findings demonstrate the potential of the fungal endophytes from *C. castaneus* spines as biocontrol agents.

## 1. Introduction

Endophytic fungi are ubiquitous and found in almost all plant parts, including stems, leaves, and roots, and colonize the host plants without causing any disease symptoms throughout their life cycle [1]. These microorganisms have shown the potential to enhance host resistance to pathogens and pests as well as tolerance to abiotic stress [2]. Bilal et al. (2008) [3] reported that endophytic *Aspergillus fumigatus* and *Fusarium proliferatum* produce growth regulators and promote plant growth under abiotic conditions. Some endophytic fungi have been reported to improve plant growth and reduce the severity of plant diseases; therefore, these fungi have the potential to be used in plant disease management strategies [4]. For example, fungal endophytes from cocoa (*Theobroma cacao*) inhibit the growth of several major pathogens of the crop [5]. Endophytic fungi may be antagonistic and inhibit the growth of other fungi, and many have been reported as potential biocontrol agents [5,6]. Biological control using endophytic fungi is an alternative method for sustainable plant disease management and contributes to environmental conservation.

Plants use several sharp structures, such as spines, thorns, and prickles, for defense. Spines are modified leaves, whereas thorns are a modification of branches, and prickles result from the outgrowth of cortical tissues in the bark [7]. *Calamus castaneus* Griff. is a common rattan species that grows in the Malaysian tropical rainforest and is classified in the palm family, Palmae or Areceae. *Calamus castaneus* is recognized by its yellow-based spines, which cover the stems and the middle part of the upper leaves. The spines are arranged as a single line on the stem, while at the bottom of the leaves, the spines are arranged in two parallel lines [8]. These sharp structures may harbor various types of fungi as the presence of endophytic fungi, particularly dermatophytes in spines, thorns, and prickles, has been reported by Halpern et al. (2011) [9]. As *C. castaneus* is common and relatively easy to find in the forests, studying the presence of endophytic fungi in the spines of this rattan species is of interest. Novel endophytic fungal isolates that have the potential to be developed as biocontrol agents against several plant pathogenic fungi might also be recovered from spines of *C. castaneus*. As there is a lack of information on the fungal endophytes from spines, the objectives of this study were to determine the occurrence of endophytic fungi in the spines of *C. castaneus* and identify the endophytic fungi through molecular methods. The antagonistic activity of the fungal endophytes from the spines to inhibit growth of several plant pathogenic fungi was also tested using a dual culture method. Knowledge on the endophytic fungal community in spines of *C. castaneus* contributes to in-depth information on the occurrence of fungal endophytes in various plant parts as well as identifying potential biocontrol agents against plant pathogens.

## 2. Materials and Methods

### 2.1. Sample Collection and Isolation of Endophytic Fungi

**Figure 1:**
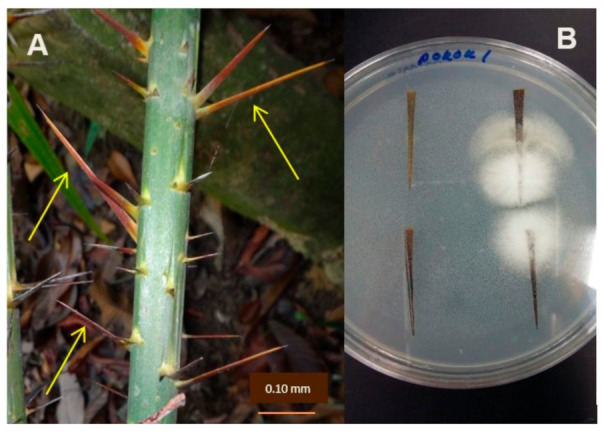
*Calamus castaneus* spines (yellow arrow) and isolation of endophytic fungi. (**A**) Spines on stem of rattan palm (*C. castaneus*). (**B**) Mycelia growth from the spines.

The spines of *C. castaneus* were randomly collected from rattan trees found in three rainforests, in two states of the Peninsula Malaysia, namely in Bukit Panchor State Park, Penang (5.1602° N, 100.5480° E); Segari Melintang Forest Reserve, Perak (4°18–20′ N, 100°34–36′ E); and Belum Rainforest, Gerik, Perak (5°34 58.34′ N, 101°15 30.7′ E). The spines were kept in an envelope and transported to the laboratory. The spines were placed in a beaker, covered with a net cloth, and placed under running tap water overnight to remove any debris, dirt, and epiphytes adhered to the surface. Thereafter, the spines were surface sterilized by soaking in 70% ethanol for 5 min, followed by 5% sodium hypochlorite (NaOCl) for 5 min. Then, the samples were washed with sterile distilled water three times for 2 min and blotted dry using sterile filter papers to remove excess water. The sterilized spines were plated onto potato dextrose agar (PDA, HiMedia Laboratory, Maharashta, India) plates and incubated at room temperature (27 ± 1 °C) until there was visible mycelial growth from the spine tissues (Figure 1). Sixty spine samples were used for isolation.

The efficiency of the surface sterilization technique was determined using an imprint method [1]. The surface sterilized spines were imprinted or dabbed on the surface of a PDA plate and the plate was incubated at room temperature. Surface sterilization is considered effective if no fungal colony grows on the imprint plate. Mycelia growing from the spine tissue were sub cultured onto new PDA plates. A pure culture of the isolate was obtained using the spore suspension method and the plates were incubated at room temperature for seven days.

The fungal isolates were sorted into their respective groups or genera based on the appearance of the colonies and microscopic characteristics.

### 2.2. DNA Extraction and PCR Amplification

The fungal isolates were grown in potato dextrose broth and incubated at room temperature for six days. Mycelia were harvested and ground with liquid nitrogen in a sterile mortar and pestle to a fine powder. The DNeasy^®^ Plant Mini kit (Qiagen, Hilden, Germany) was used to extract genomic DNA, according to the manufacturer’s instructions. 

The internal transcribed spacer (ITS) region was used to identify all endophytic fungal isolates recovered from the spines except *Xylaria*. The primers used were ITS1 and ITS4 [10]. After amplification of the ITS, species identity was obtained based on the basic local alignment search (BLAST) and a combination of at least two genes/regions was used for further confirmation of the species (Table 1). However, for several fungal genera, the analysis of the ITS region was not sufficient to differentiate closely related species. 

PCR reactions were prepared in a total volume of 50 µL containing 8 µL of 5X Green GoTaq^®^ Flexi Buffer, 8 µL of 25 mM MgCl_2_, 1 µL of 10 mM dNTP mix, 8 µL each of 5 µM forward and reverse primers, deionized distilled water, 0.3 µL of 5 U/µL GoTaq^®^ DNA Polymerase (Promega, Madison, WI, USA), and 0.6 µL of DNA template. EconoTaq^®^ Plus Green 2× Master Mix reagent (Middleton, WI, USA) was used to amplify β-tubulin and ACT. The PCR reaction was prepared in a total volume of 50 µL containing 25 µL EconoTaq^®^ Plus Green 2× Master Mix, 0.5 µL each of the forward and reverse primers (100 µM), 1 µL of DNA template, and deionized distilled water. The amplification was performed in a thermal cycler (Bio-Rad MyCycler PCR System version 1.065) programmed to 85 s at 94 °C, 35 s at 95 °C for 35 cycles, 55 s at 59 °C, 90 s at 72 °C, and a final 10 min extension at 72 °C. A 1% agarose gel (Promega, Middleton, WI, USA) was used to detect the PCR products in 1 ×Tris-Borate-EDTA (TBE) buffer stained with FloroSafe DNA stain (Axil Scientific, Singapore). PCR products were sent to a service provider for Sanger DNA sequencing.

### 2.3. Molecular Identification and Phylogenetic Analysis

The DNA sequences were aligned manually and edited using the Molecular Evolution Genetic Analysis version 7 (MEGA7 version 7) [18]. Forward and reverse sequences were aligned with ClustalW using pairwise alignments. The aligned forward and reverse sequences were edited when necessary to form a consensus sequence. For species identity, a BLAST search was used to analyze the number of bases and determine the maximum identity of the consensus sequences from the GenBank database. 

A phylogenetic analysis was also conducted, particularly for species that are known to belong to a species complex or for isolates whose ITS sequences cannot be used to confidently identify the isolates to the species levels. Multiple sequence alignments were generated and used to construct phylogenetic trees based on combined sequences. A maximum likelihood (ML) tree was constructed with 1000 bootstraps replicates. The heuristic method used in ML was the nearest neighbor interchange (NNI) and the initial tree for ML was generated automatically. The best model for ML tree was determined from the model search with number of discrete gamma categories 5. The results show that the Kimura 2 parameter model was the best model. Missing data or gaps were treated as complete deletion.

### 2.4. Antagonistic Activity

The ability of the fungal endophytes to inhibit the mycelial growth of several plant pathogenic fungi was determined with a dual culture method using PDA. Several endophytic fungi from *C. castaneus* spines were selected to assess their antagonistic activity against several plant pathogenic fungi. The endophytic fungi were chosen based on fungal genera or species that have been reported as antagonists against plant pathogens, such as *Xylaria cubensis*, *Penicillium indicum, Penicillium oxalicum, Trichoderma harzianum*, and *Trichoderma koningiopsis*. Endophytic fungal species that have not been reported as antagonists were also tested, namely *Endomelanconiopsis endophytica*, *Neopestalotiopsis saprophytica*, *Colletotrichum endophytica*, *Colletotrichum siamense*, *Colletotrichum boninense*, *Diaporthe arengae*, *Diaporthe tectonae*, *Diaporthe* cf. *nobilis*, and *Diaporthe* cf. *heveae*.

Selected plant pathogenic fungi were obtained from the culture collection at the Plant Pathology Laboratory, School of Biological Sciences, Universiti Sains Malaysia, Penang, Malaysia. The pathogenic fungi included two anthracnose chili pathogens, *C. truncatum* and *C. scovellei*; two pathogens that cause dragon fruit stem rot, *Fusarium proliferatum* and *F. fujikuroi*; and *F. solani* and *F. oxysporum*, which are associated with crown disease in oil palm. Four pathogens associated with mango diseases were also included: *Lasiodiplodia theobromae* and *Pestalotiopsis mangiferae*, which are the causal pathogens of the mango leaf spot, and *L. pseudotheobromae* and *D. pascoei*, which cause mango stem-end rot.

A combination of the endophytic fungi and plant pathogenic fungi tested in dual culture test is shown in Table 2. A control plate harbored only plant pathogenic fungi without the endophytes. Mycelial plugs (5 mm) of the pathogen and endophyte were cultured 6 cm apart. The plates and three replications were incubated at room temperature for seven days. The experiment was repeated twice.

After seven days, the percentage of the pathogen growth inhibition (PGI) was calculated according to the method described by Skidmore and Dickinson (1976) [19]:

PGI (%) = (R1 − RI2/R) × 100.

R1—radial growth of plant pathogenic fungi in control plate.
R2—radial growth of plant pathogenic fungi in dual culture plate.

R1 was measured from the point of inoculation to the pathogen colony margin on the control plate and R2 was measured from the point of inoculation to the colony margin on the dual culture plate in the direction of the endophytes. 

Statistical analysis of the PGI value was performed using ANOVA in SPSS statistical software version 24. Interactions between plant pathogens and endophytic fungi were assigned in a range of interactions from types A to E, according to the interactions described by Skidmore and Dickinson (1976) [19]. Type A interactions occurred when the pathogens and endophytic fungi displayed intermingling growth; type B interactions represented the overgrowth of pathogens by endophytic fungi; type C interactions represented the overgrowth of endophytic fungi by pathogens; type D interactions represented mutual inhibition with a clear inhibition zone at small distance (<2 mm); and type E interactions represented mutual inhibition with a clear inhibition zone at a greater distance (>2 mm).

## 3. Results

### 3.1. Molecular Identification

A total of 108 isolates of endophytic fungi comprising 21 genera with 40 species were recovered from the *C. castaneus* spines (Table 3). Fungi isolated from the spines were confirmed as endophytes as no fungal growth on the imprinted plates was observed. The imprint method was used as an indication that the epiphytes from the surface of the spines had been removed. A successful and correct procedure of surface sterilization removes epiphytes from the surface of the spines, which results in no fungal growth and must be used in all studies concerning endophytes [20,21]. 

Endophytic fungal species recovered from *C. castaneus* spines identified using ITS and other additional markers are shown in Table 2. Most of the isolates were successfully identified to the species levels except for three isolates of *Diaporthe*. The most common isolates recovered from the spines were *Colletotrichum* spp. (*n =* 19) and *Diaporthe* spp. (*n =* 18), followed by *Phyllosticta* spp. (*n =* 11), *Xylaria* sp. (*n =* 9), *Trichoderma* spp. (*n =* 7), *Helminthosporium* spp. (*n =* 7), *Penicillium* spp. (*n =* 6), *Fusarium* spp. (*n =* 6), *Neopestalotiopsis* spp. (*n =* 3), *Arthrinium* sp. (*n =* 3), *Cyphellophora* sp. (*n =* 2), *Cladosporium* spp. (*n =* 2), *Curvularia* sp. (*n =* 1), *Bionectria* sp. (*n =* 1), *Acremonium* sp. (*n =* 1), and six species of non-sporulating fungi.

**Figure 2:**
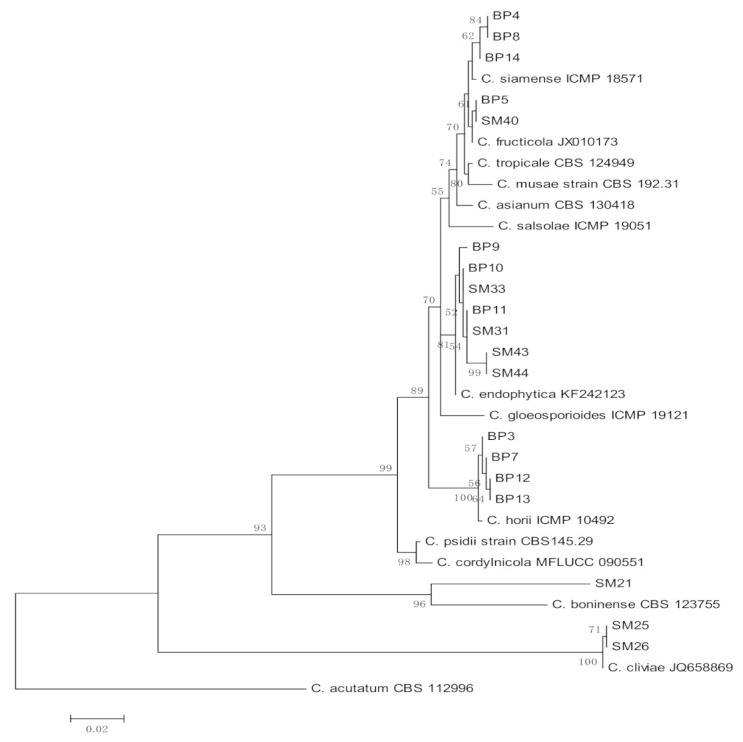
Maximum likelihood tree inferred from combined sequences of internal transcribed spacer (ITS) and GAPDH of *Colletotrichum* isolates from *C. castaneus* spines with bootstrap values higher than 50% are shown next to the branches.

Six species of *Colletotrichum* were identified using ITS and GAPDH sequences, namely *C. horii* (*n =* 4), *C. siamense* (*n =* 3), *C. fructicola* (*n =* 2), *C. cliviae* (*n =* 2), *C. endophytica* (*n =* 7), and *C. boninense* (*n =* 1) (Table 3). All the species identified are members of the *C. gloeosporioides* species complex. In addition to ITS, the *GAPDH* gene was included as an additional marker as the gene is among the most effective secondary markers to distinguish species in the genus *Colletotrichum*. Moreover, *GAPDH* is the easiest gene to amplify and sequence [22,23]. The phylogenetic analysis showed that isolates from the same species were grouped in the same clade as their epitype strains (Figure 2), which confirmed the identity of the endophytic *Colletotrichum* species obtained from *C. castaneus* spines.

**Figure 3:**
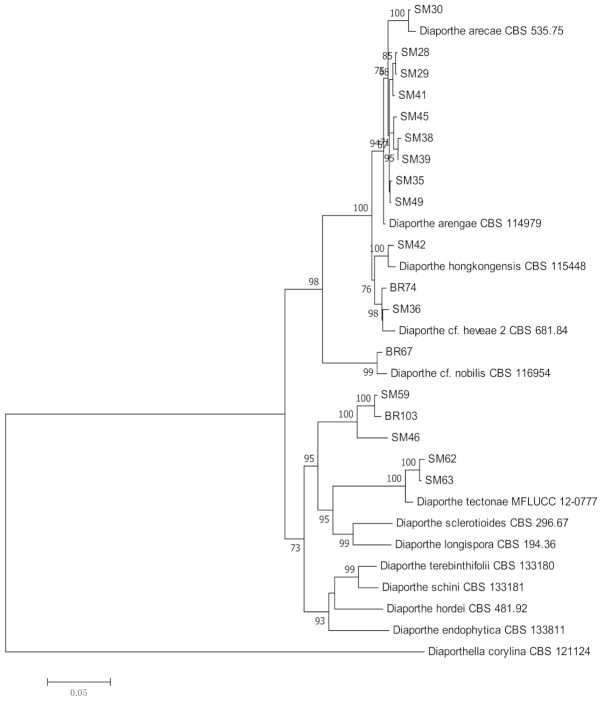
Maximum likelihood tree inferred from combined sequences of ITS, TEF-1α, and β-tubulin of *Diaporthe* isolates from *C. castaneus* spines with bootstrap values higher than 50% are shown next to the branches.

Based on phylogenetic analysis of the combined ITS, TEF-1α, and β-tubulin sequences, 18 isolates of *Diaporthe* spp. were phylogenetically identified as *D. arengae* (*n =* 8), *D. hongkongensis* (*n =* 1), *Diaporthe* cf. *heveae* 2 (*n =* 2), *D.* cf. *nobilis* (*n =* 1), *D. arecae* (*n =* 1), *D. tectonae* (*n =* 2), and *Diaporthe* spp. (*n =* 3). In the ML tree, isolates of the same species were grouped together with their epitype strains (Table 2, Figure 3). 

**Figure 4:**
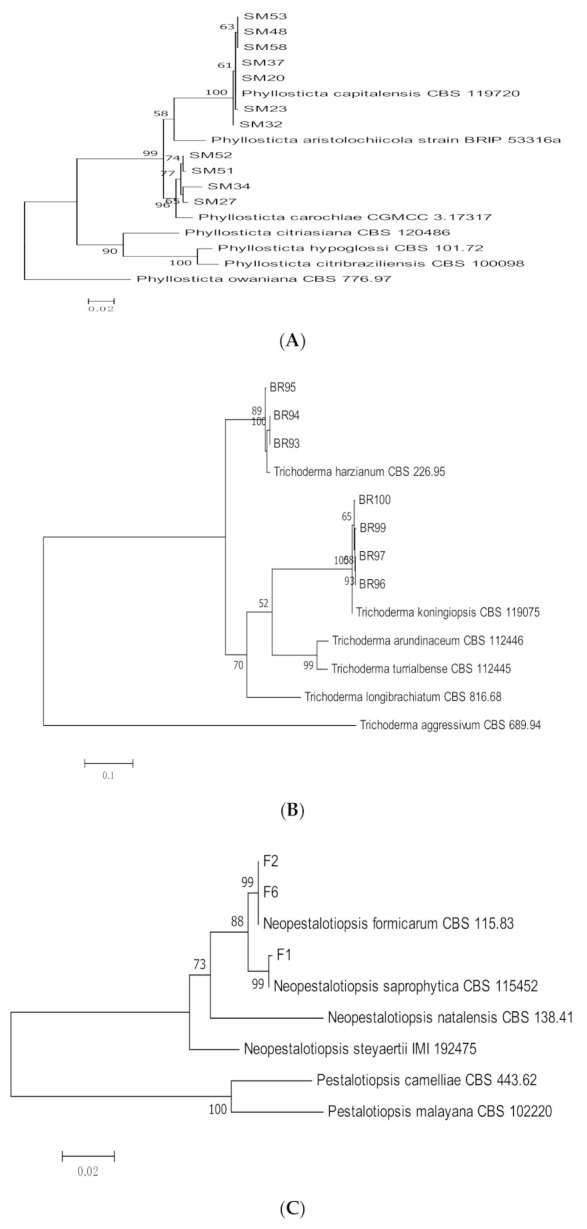
Maximum likelihood tree inferred from combined sequences of ITS and TEF-1α for (**A**) *Phyllosticta* spp., (**B**) *Trichoderma* spp., and (**C**) *Neopestalotiopsis* spp. from *C. castaneus* spines with bootstrap values higher than 50% are shown next to the branches.

Endophytic isolates of *Phyllosticta*, *Trichoderma*, and *Neopestalotiopsis* were identified through molecular methods using ITS and TEF-1α sequences (Table 3, Figure 4A–C). Isolates of *Phyllosticta* were identified as *P. capitalensis* (*n =* 7) and *P. carochlae* (*n =* 4). Seven isolates of endophytic *Trichoderma* were identified as *T. harzianum* (*n =* 3) and *T. koningiopsis* (*n =* 4). Two species of endophytic *Neopestalotiopsis, N. saprophytica* (*n =* 1) and *N. formicarum* (*n =* 2) were also isolated from *C. castaneus* spines.

**Figure 5:**
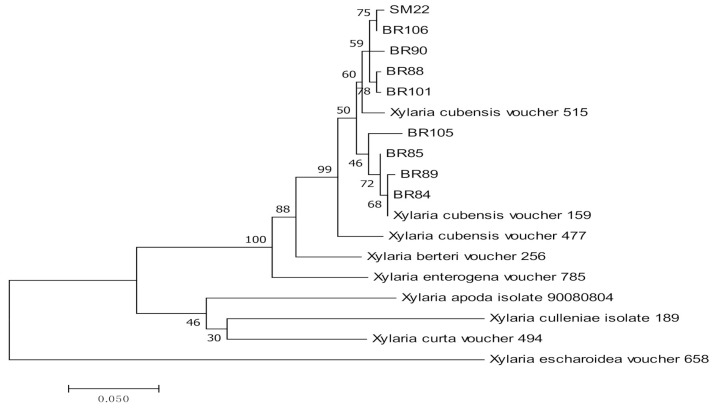
Maximum likelihood tree inferred from combined sequences of β-tubulin and ACT of *X. cubensis* from *C. castaneus* spines with bootstrap values higher than 50% are shown next to the branches.

Nine isolates of the endophytic *X. cubensis* were identified using β-tubulin and ACT sequences (Table 3, Figure 5). 

**Figure 6:**
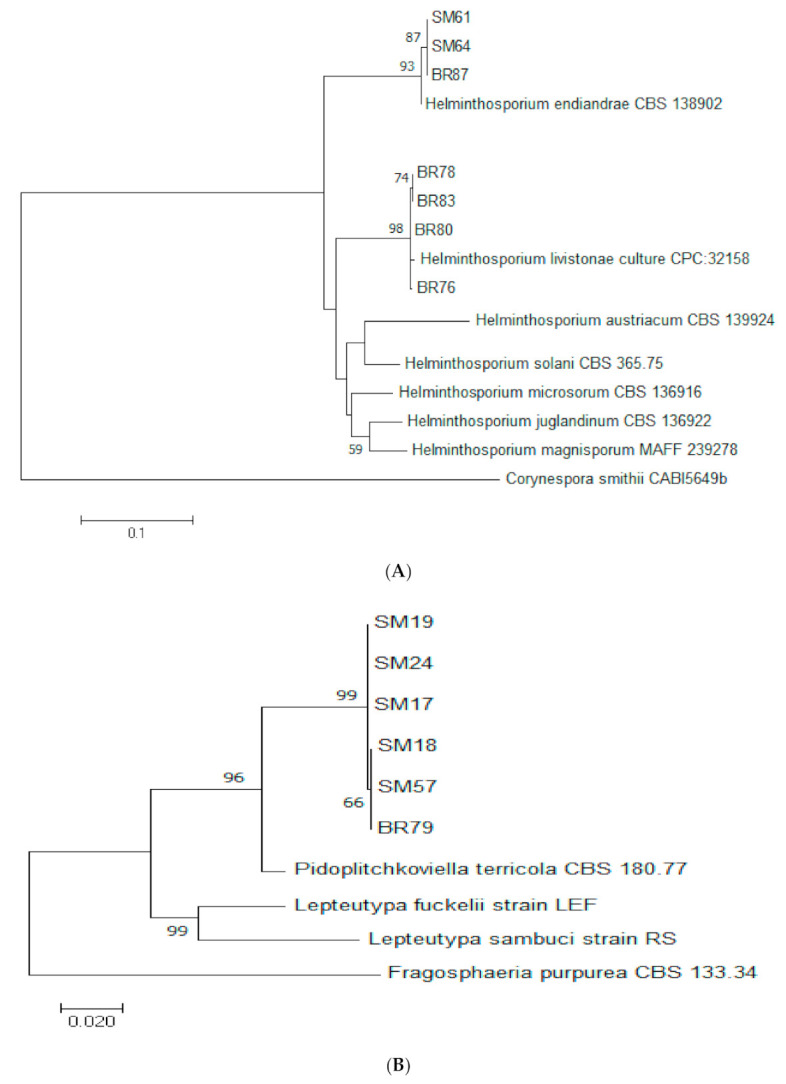
Maximum likelihood tree inferred from combined sequences of ITS and LSU for (**A**) *Helminthosporium* spp. and (**B**) *Pidoplitchkoviella terricola* from *C. castaneus* spines with bootstrap values higher than 50% are shown next to the branches.

Based on ITS and LSU sequences, endophytic isolates of *Helmintosporium* were identified as *H. livistonae* (*n =* 5) and *H. endiandrae* (*n =* 2) (Table 2, Figure 6A). Isolates of *Pidoplitchkoviella terricola* (*n =* 6) were identified using ITS and LSU sequences. The endophytic *P. terricola* isolates were clustered in the same main clade as the reference strain (CBS 180.77) but the isolates formed a separate sub-clade (Figure 6B), which might indicate that the isolates represent different phylogenetic strains of the species.

**Figure 7:**
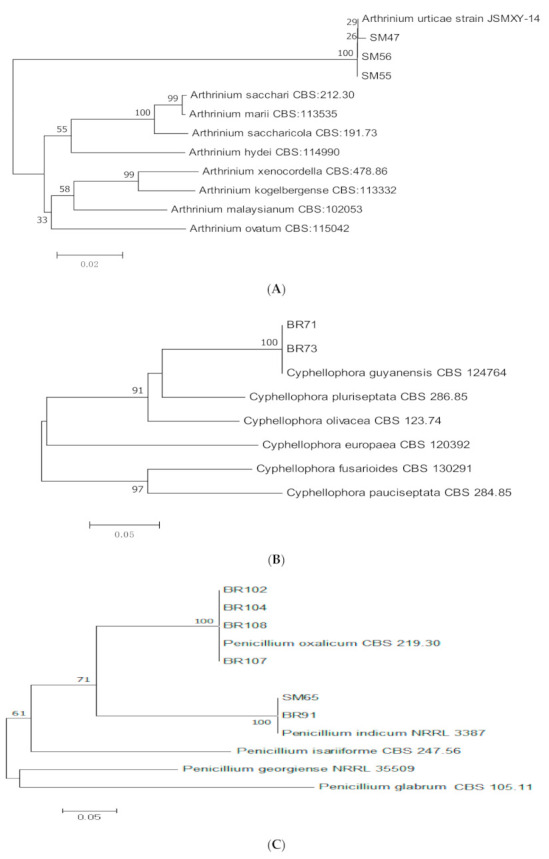
Maximum likelihood tree inferred from combined sequences of ITS and β-tubulin for (**A**) *Arthrinium urticae,* (**B**) *Cyphellophora guyanensis*, and (**C**) *Penicillium* spp. from *C. castaneus* spines with bootstrap values higher than 50% are shown next to the branches.

Based on ITS and β-tubulin sequences, isolates of endophytic *Arthrinium urticae* (*n =* 3)*, Cyphellophora guyanensis* (*n =* 2), and two species of *Penicillium*, *P. indicum* (*n =* 2) and *P. oxalicum* (*n =* 4) were identified (Table 3, Figure 7A–C).

**Figure 8:**
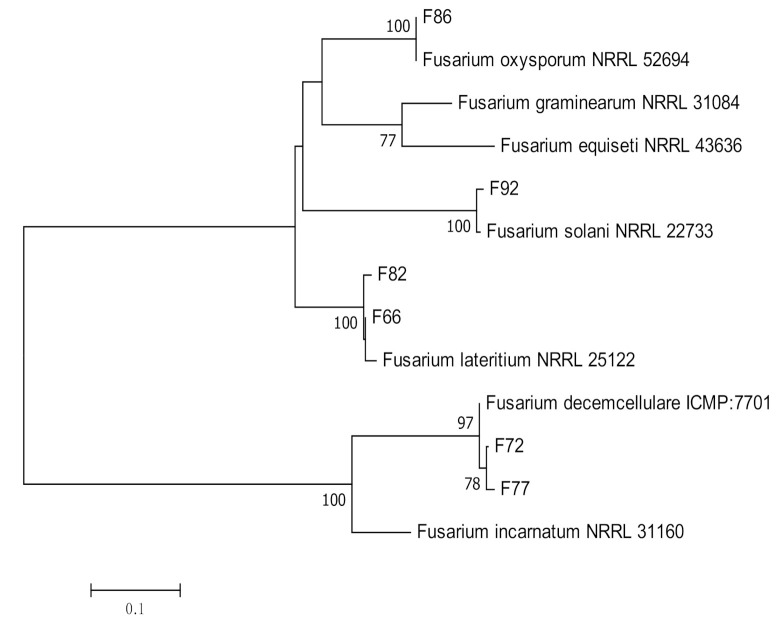
Maximum likelihood tree inferred from combined sequences of TEF-1α and β-tubulin of *Fusarium* spp. from *C. castaneus* spines with bootstrap values higher than 50% are shown next to the branches.

**Figure 9:**
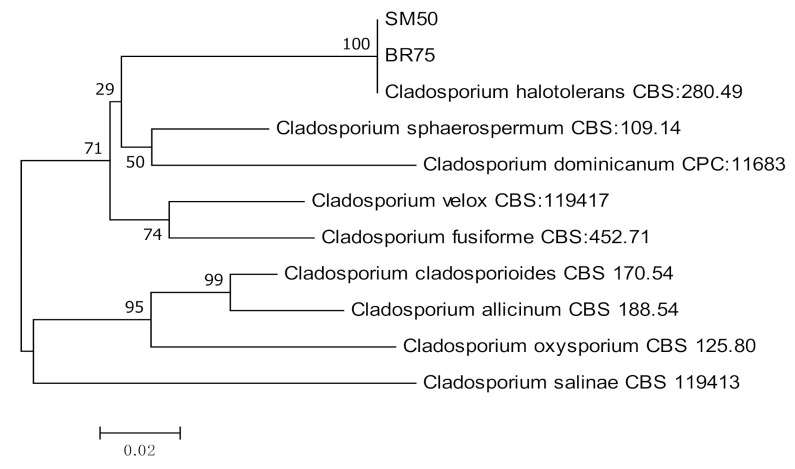
Maximum likelihood tree inferred from combined sequences of ITS and ACT of *C. halotolerans* isolates from *C. castaneus* spines with bootstrap values higher than 50% are shown next to the branches.

Four species of endophytic *Fusarium*, *F. lateritium* (*n =* 2), *F. decemcellulare* (*n =* 2), *F. oxysporum* (*n =* 1), and *F. solani* (*n =* 1) were identified using TEF-1α and β-tubulin (Table 3, Figure 8). Two isolates of *Cladosporium halotolerans* were identified using ITS and ACT sequences (Table 3, Figure 9).

**Figure 10:**
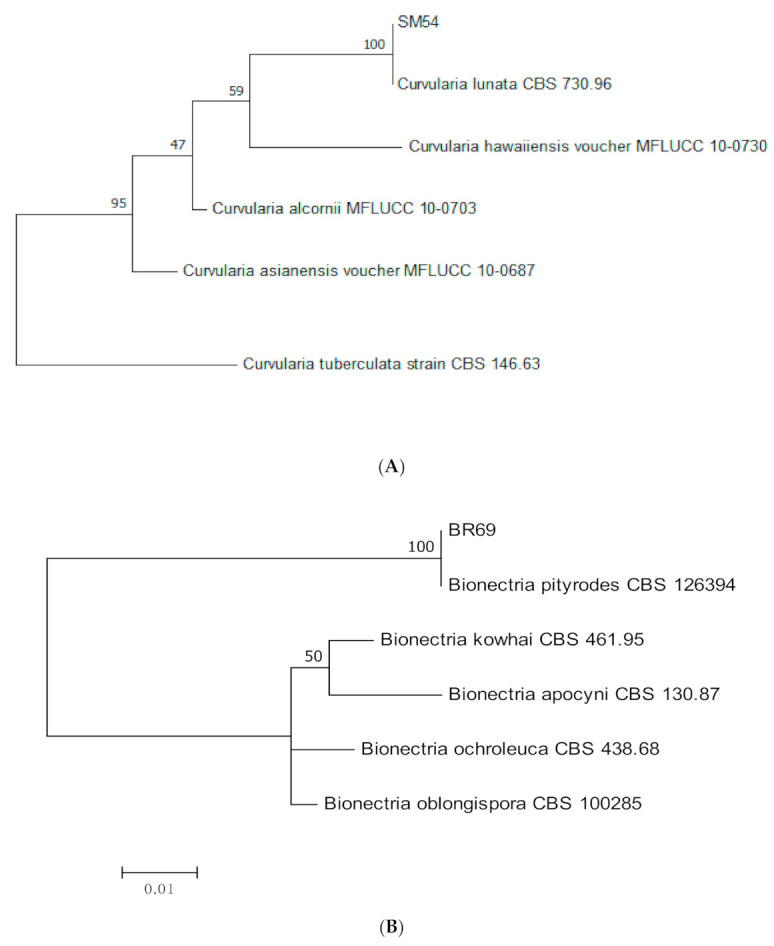

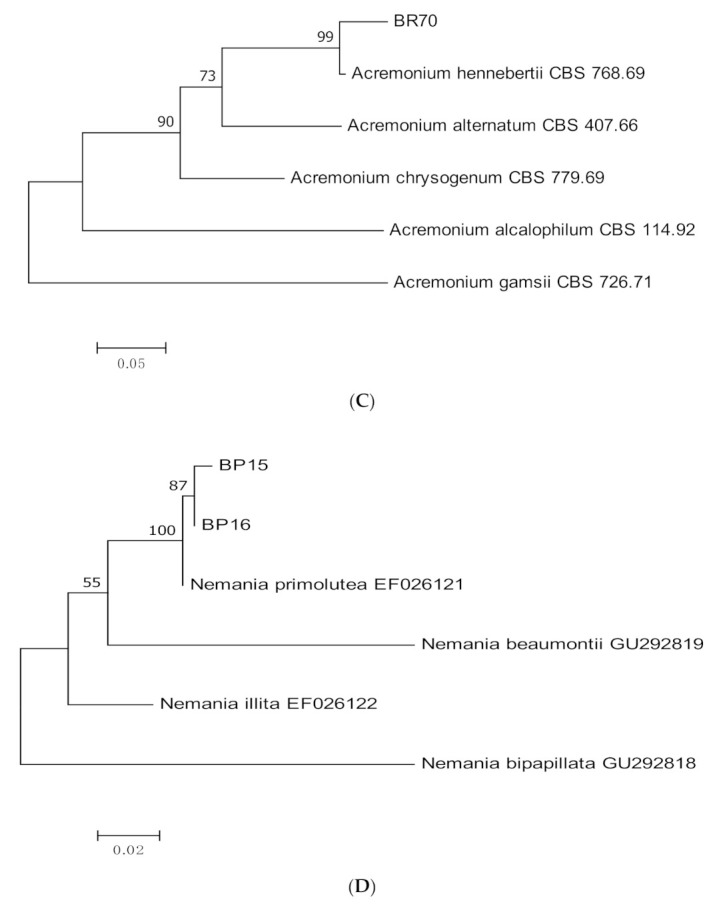

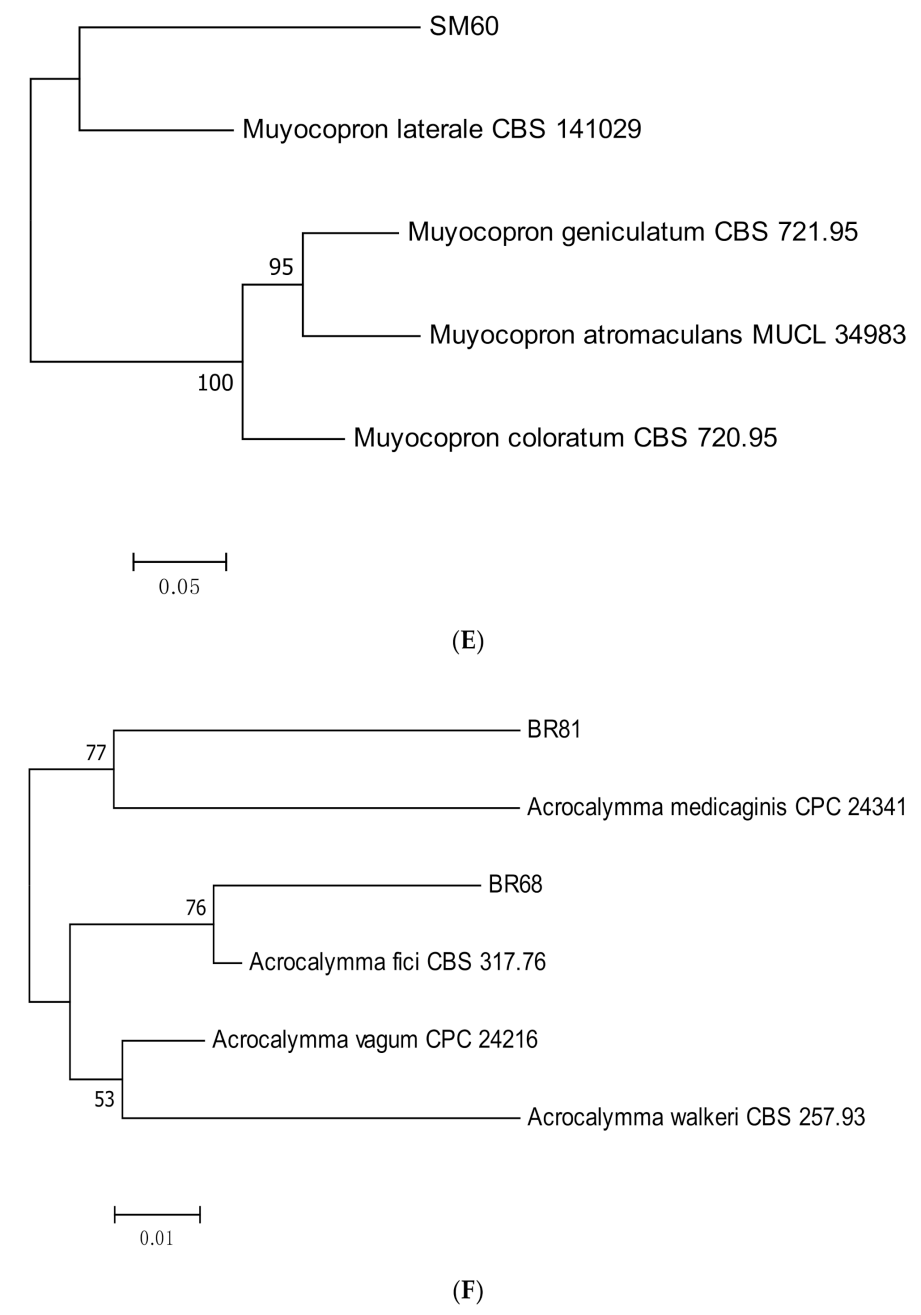

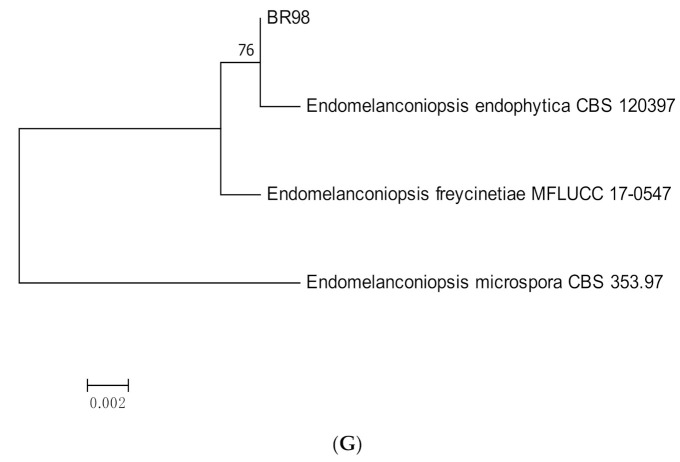
(**A**–**G**) Maximum likelihood tree inferred from combined sequences of ITS for (**A**) *Curvularia lunata*, (**B**) *Bionectria pityrodes* (**C**) *Acremonium hennebertii*, (**D**) *Nemania primolutea*, (**E**) *Muyocopron laterale*, (**F**) *Acrocalymma* spp., and (**G**) *Endomelanconiopsis endophytica* from *C. castaneus* spines of with bootstrap values higher than 50% are shown next to the branches.

Several species of the endophytic fungi were identified using ITS sequences (Table 3, Figure 10A–G), namely *Curvularia lunata* (*n =* 1), *Bionectria pityrodes* (*n =* 1), *Acremonium hennebertii* (*n =* 1), *Nemania primolutea* (*n =* 2), *Muyocopron laterale* (*n =* 1), *Acrocalymma fici* (*n =* 1)*, Acrocalymma medicaginis* (*n =* 1), and *Endomelanconiopsis endophytica* (*n =* 1).

### 3.2. Antagonistic Activity

In general, most of the endophytic fungi from *C. castaneus* spines inhibited mycelial growth of the plant pathogenic fungi tested (Table 4). Only three species of *Diaporthe, D.* cf. *nobilis*, *D.* cf. *heveae*, and *D. tectonae*, as well as two isolates of *X. cubensis* did not show antagonistic activity against *L. theobromae* and *L. pseudotheobromae* (Table 4). Both pathogens overgrew the endophytic fungi as *L. theobromae* and *L. pseudotheobromae* are fast growing fungi able to compete for space and nutrients.

Based on the observation of the dual culture plates, the most common interactions between the fungal endophytes and plant pathogenic fungi were type D interaction, which is mutual inhibition with a clear inhibition zone (<2 mm).

**Figure 11:**
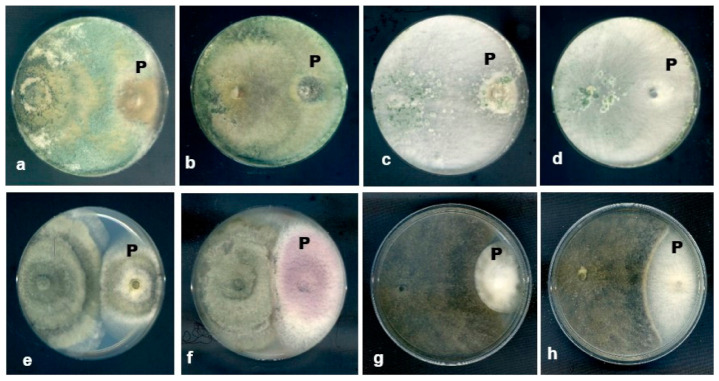
Antagonistic activity of endophytic fungi against several plant pathogenic fungi (P) on dual culture plates. *T. harzianum* overgrew (**a**) *C. scovellei* and (**b**) *C. truncatum*; *T. koningiopsis* overgrew (**c**) *C. scovellei* and (**d**) *C. truncatum*; *E. endophytica* moderately inhibited (**e**) *L. theobromae* and (**f**) *F. oxysporum*; and *D. tectonae* moderately inhibited (**g**) *C. scovellei* and (**h**) *F. oxysporum.*

Both endophytic *T. harzianum* and *T. koningiospsis* overgrew the pathogens on the 7th day of incubation. *Endomelanconiopsis endophytica* and *D. tectonae* moderately inhibited all tested plant pathogens (Figure 11). The results showed that the pathogens were lysed and subsequently killed as no growth was observed when the hyphae from the contact point of both fungi in the dual culture test were transferred onto PDA. A high percentage of growth inhibition was shown by the endophytic *T. harzianum* and *T. koningiopsis* that inhibited the mycelial growth of all tested plant pathogens (Table 4).

## 4. Discussion

A total of 108 isolates of endophytic fungi comprising 21 genera with 40 species were recovered from *C. castaneus* spines. The results showed that endophytic fungi residing in the spines are mostly Ascomycetes, class Sardariomycetes, order Glomerellales (*Colletotrichum*), Diaporthales (*Diaporthe*), Xylariales (*Xylaria*), Hypocreales (*Trichoderma*, *Fusarium*), as well as several other classes and orders. The present study demonstrated that endophytic fungi isolated residing in *C. castaneus* spines may be considered as cosmopolitan fungal isolates.

The endophytic fungi from *C. castaneus* spines were identified using ITS and other suitable markers. Despite the advantages of the ITS region for fungal identification, the region may not be useful to distinguish species in a species complex or closely related species, such as *Colletotrichum* and *Diaporthe*. This may be due to lower sequence variation in many closely related species, the presence of sequence heterogeneity among the ITS copies, and the inability of some groups of fungi to amplify the ITS region resulting in poor sequencing success [24,25]. Hence, several genes were also used to accurately identify the fungal isolates and for phylogenetic analysis. The gene chosen depends on the fungal genera; *TEF-1α*, β-tubulin, *GAPDH*, and *ACT* genes were used in this study. Introns in protein-coding genes are highly variable, which make them useful for species identification and phylogenetic analyses. Several of these genes are considered secondary barcode markers with adequate intra- and interspecies variation often used as part of identification using multiple gene phylogeny [25].

Based on the genera and species identified, most of the fungal endophytes isolated from the spines of *C. castaneus* have been isolated from other plants and plant parts. The genera *Colletotrichum*, *Diaporthe*, *Xylaria*, *Phyllosticta*, *Trichoderma*, *Penicillium*, and *Fusarium* are common endophytes. These genera have been reported in various types of plants, including a medicinal plant (*Carapa guianensis*) [26], palms (*Livistona chinensis* and *Ptychosperma macarthuri*) [27,28], coffee berries (*Coffea arabica*) [29] and mangrove (*Rhizophora stylosa*) [30].

The endophytic fungal species from genera *Colletotrichum*, *Trichoderma, Penicillium, Phomopsis*, *Phyllosticta*, and *Xylaria* are among common fast-growing culturable fungi, which might be one of the reasons these genera were mostly recovered as endophytic fungi from the spines. Moreover, the methods used in this study were culture-dependent methods of which only culturable isolates were recovered from the spines. In culture-dependent methods, several growth parameters including temperature, light, nutrient, and aeration contribute to the growth of the endophytic fungi [31]. By using culture-dependent methods, fast-growing fungal isolates commonly inhibit the growth of slow-growing isolates and thus many fast-growing fungi were recovered [32]. Unculturable endophytic fungi could not grow or were difficult to grow on culture media. Thus, unculturable endophytic fungi are commonly analyzed using culture-independent methods such as denaturing gradient gel electrophoresis and high-throughput sequencing methods [33,34]. These methods can directly amplify endophytic fungi residing in the plant tissues.

*Colletotrichum* spp. (*n =* 19) and *Diaporthe* spp. (*n =* 18) were the most common endophytes isolated from *C. castaneus* spines. Species from both genera have been reported as endophytes in the roots, leaves, and stem of several plants, including mangrove tree leaves (*Acanthus ebracteatus* and *Phoenix paludosa*) [35], leaves of *Sapindus saponaria* [36], and twigs of a woody tree (*Acer truncatum*) [37]. Therefore, the endophytic fungal species from both genera isolated from *C. castaneus* spines are similar to those previously reported from other types of plants that harbor fungal endophytes [35,36,37].

Although numerous endophytic species from *C. castaneus* spines are common endophytes, several species have not been reported as endophytes from any plant. These endophytes are *P. carochlae, P. indicum, Arthrinium urticae*, *C. guyanensis, A. hennebertiiennebertii*, and *P. terricola.* Among these endophytic fungi, *P. terricola* is a rare species and was only reported in the rhizosphere of *Quercus rubra* in Ukraine [38] and from earthworm casts in Domica Cave, Slovakia [39].

Dermatophytes of animals and humans have been reported from spines, thorns, and prickles [40]. Dermatophytes causing subcutaneous mycosis and infection may occur by inoculation of the dermatophytes into subcutaneous tissues by penetration of spines and thorns [41,42]. Among the dermatophytes from plants, *Fonsecaea pedrosoi* was reported in thorns of *Mimosa pudica* isolated from the site of infection [43]. *Cladophialophora carrionii* has also been isolated from plants. Another dermatophyte, *Sporothrix schenckii*, is commonly transmitted through a prick from roses [44,45]. However, in the present study, dermatophytes were not recovered from *C. castaneus* spines, which might be due to different host plants, environmental conditions, and geographical location. These factors may contribute to the endophytic fungi occurrence and diversity in the host plant [46,47].

An antagonistic activity assay was conducted to assess the ability of the fungal endophytes from *C. castaneus* spines to be used as antagonists that inhibit the growth of plant pathogens. Among the endophytic fungi recovered from *C. castaneus* spines, *T. harzianum*, and *T. koningiospsis* highly inhibited growth of all tested plant pathogens. Other endophytic fungi tested produced low to moderate inhibition. The results of the present study indicated endophytic *T. harzianum* and *T. koningiopsis* showed strong antagonistic effects against all the pathogens tested and successfully inhibited the growth of the pathogens. *Trichoderma harzianum* has been reported to inhibit growth of *C. truncatum*, causal pathogen of strawberry anthracnose [48], and mango anthracnose [49]. So far, there are no reports on antagonistic activity of *T. koningiopsis* against anthracnose pathogens, but this species has strong antagonistic activity against *F. oxysporum*, *Rhizoctonia solani*, and *Botrytis cinerea* that infected tomato and cucumber seedlings [50]. *Trichoderma koningiopsis* was also reported as strong antagonistic fungus, showing 85% growth inhibition of *Calonectria pseudonaviculata* causing blight of boxwood plant [51].

Several reports are available on the antagonistic activity of *T. harzianum* against plant pathogenic *Fusarium* spp. *Trichoderma harzianum* inhibited growth of *F. proliferatum*, causing basal rot of onion bulb [52] and stalk rot of maize [53] as well as inhibiting growth of *F. solani*, causal pathogen of root rot of olive tree [54]. As for *T. koningiopsis*, this fungus exhibited strong antagonistic activity against *F. proliferatum,* causal pathogen of soybean damping-off [55].

As one of the effective antagonistic fungi, *Trichoderma* spp. have several mechanisms of inhibition, which include competition for space and nutrients, antibiosis by secretion of antifungal compounds, mycoparasitism, and induced resistance [56]. These mechanisms may occur with *T. harzianum* and *T. koningiospsis* as both grew faster than the pathogens.

*Endomelanconiopsis endophytica* and *D. tectonae* may also be considered as effective antagonistic fungi. Both endophytic fungi moderately inhibited the mycelial growth of all tested plant pathogens except for *L. theobromae* and *L. pseudotheobromae*, whereby both pathogens grew faster than the endophytes. The inhibition mechanisms might be similar to that of *Trichoderma* spp., in which the mycelial growth of the tested pathogens was inhibited by competition, antibiosis, or mycoparasitism.

Antagonistic activity of *E. endophytica* against other plant pathogenic fungi has not been reported, but in a study by Ferreira et al. (2015) [26], the extract of this endophytic fungus displayed trypanocidal activity against amastigote forms of *Trypanosoma cruzi*. For endophytic *D. tectonae*, this fungus moderately inhibited growth of *Phytopthora palamivora,* pathogen of cocoa black pod [57].

Endophytic fungi residing in the spines exhibited antagonistic activity, indicating their ability to produce bioactive compounds. These bioactive compounds may be involved in defense mechanisms against pathogen infections, chemical defense [6,58], and adaption and survival in the host plant [26].

Various groups of chemical compounds were produced by endophytic fungi including alkaloids, chinones, cytochalasins, depsipeptides, flavanoids, furandiones, isocoumarins, peptides, phenols, perylene derivatives, quinines, steroids, terpenoids, and xanthones [59,60,61,62]. Several of these bioactive compounds exhibited antifungal activity against plant pathogenic fungi. For example, koninginins recovered from *T. koningiopsis* have been reported to inhibit growth of *F. solani*, *F. oxysporum*, and *Alternaria panax* [63]. *Trichoderma harzianum* ability to reduce pathogens of stored kiwi fruits, and Fusarium wilt of cucumber was due to a compound identified as pyrone 6-pentyl-2H-pyran-2-one (6-PP) [63,64]. There are in fact various types of compounds identified from endophytic fungi that exhibited antifungal activity against fungal pathogens [65,66,67,68].

As a conclusion, a total of 108 isolates of endophytic fungi were isolated from *C. castaneus* spines and 40 species were identified. The results demonstrate that *C. castaneus* spines harbor diverse groups of endophytic fungi with an antagonistic activity against several plant pathogenic fungi. Among the endophytic fungi, *T. harzianum* and *T. koningiopsis* inhibited all plant pathogens tested with a high percentage of inhibition. The antagonistic activity against plant pathogenic fungi indicated that the endophytic fungi have the potential to be developed for use as biocontrol agents. Therefore, further studies should be performed to detect and identify bioactive compounds produced by the endophytic fungi as well as to understand the mechanism the endophytes used to inhibit the pathogen growth. To the best of our knowledge, the present study is the first to determine the occurrence and diversity of filamentous fungi in spines of rattan palm.

## Figures and Tables

**Table 1:** Gene/regions used for the identification of endophytic fungi from *C. castaneus* spines.

Region/Gene	Primers	Sequence (5′-3′)	Fungal Genera	References
ITS	ITS 1	TCC GTA GGT GAA CCT GCG G	All fungal genera	White et al. (1990) [10]
	ITS 4	TCC GCT TAT TGA TAT GC		
*GAPDH*	GDF1	GCC GTC AAC GAC CCC TTC ATT GA	*Colletotrichum* spp.	Templeton et al. (1992) [11]
	GDR2	GGG TGG AGT CGT ACT TGA GCA TGT		
*TEF-1α*	EF1	ATG GGT AAG GAG GAC AAG AC	*Fusarium* spp.	
	EF2	GGA AGT ACC AGT GAT CAT GTT		
	EF1-728F	CAT CGA GAA GTT CGA GAA GG	*Diaporthe* spp.	O’Donnell et al. (1998) [12]
	EF1-986R	TAC TTG AAG GAA CCC TTA CC		
	EF1-728F	CAT CGA GAA GTT CGA GAA GG	*Phyllosticta* spp.	Carbone and Kohn (1999) [13]
	EF2	GGA AGT ACC AGT GAT CAT GTT	*Arthrinium* sp.	
			*Pestalotiopsis* spp.	
	EF1-728F	CAT CGA GAA GTT CGA GAA GG	*Trichoderma* spp.	
	TEF1-rev	GCC ATC CTT GGA GAT ACC AGC		
β-tubulin	T1	AAC ATG CGT GAG ATT GTA AGT	*Xylaria* sp.	
	T22	TCT GGA TGT TGG GAA TCC		
	T1	AAC ATG CGT GAG ATT GTA AGT	*Fusarium* spp.	O’Donnell and Cigelnik (1997) [14]
	T2	TAG TGA CCC TTG GCC CAG TTG		
	Bt2a	GGT AAC CAA ATC GGT GCT TTC	*Penicillium* spp.	Glass and Donaldson (1995) [15]
	Bt2b	ACC CTC AGT GTA GTG ACC CTT GGC		
	T1	AAC ATG CGT GAG ATT GTA AGT	*Cyphellophora* sp.	
	Bt2b	ACC CTC AGT GTA GTG ACC CTT GGC	*Diaporthe* spp.	
*ACT*	ACT-512F	ATG TGC AAG GCC GGT TTC G	*Xylaria* sp.	Carbone and Kohn (1999) [13]
	ACT-783R	TAC GAG TCC TTC TGG CCC AT	*Cladosporium* sp.	
LSU	LROR	ACC CGC TGA ACT TAA GC	Non-sporulating fungi	Vilgalys and Hester (1990) [16]
	LR5	TCC TGA GGG AAA CTT CG		
	V9G	TTA CGT CCC TGC CCT TTG TA	*Corynespora* spp.	De Hoog and Gerrits Van Den Ende (1998) [17]
	LR5	TCC TGA GGG AAA CTT CG		Vilgalys and Hester (1990) [16]

**Table 2:** Combination of endophytic fungi and plant pathogenic fungi tested in dual culture test.

	Endophytic Fungi								
Plant Pathogenic Fungi	*C. endophytica* (BP9)	*C. siamense* (BP14)	*C. boninense* (SM21)	*X. cubensis* (SM22)	*X. cubensis* (BR90)	*D. arengae* (SM45)	*D. tectonae* (BR62)	*D.* cf. *nobilis* (BR67)	*D.* cf. *heveae* (BR74)
*C.* *truncatum*	√	√	√	√	√	√	√	√	√
*C. scovellei*	√	√	√	√	√	√	√	√	√
*F. solani*	√	√	√	√	√	√	√	√	√
*F. oxysporum*	√	√	√	√	√	√	√	√	√
*F. proliferatum*	√	√	√	√	√	√	√	√	√
*F. fujikuroi*	√	√	√	√	√	√	√	√	√
*L. theobromae*	√	√	√	√	√	√	√	√	√
*P. mangiferae*	√	√	√	√	√	√	√	√	√
*L. pseudotheobromae*	√	√	√	√	√	√	√	√	√
*D. pascoei*	√	√	√	√	√	√	√	√	√
**Plant Pathogenic Fungi**	**Endophytic Fungi**								
	***N. saprophytica* (BP1)**	***Pen. indicum* (BR91)**		***T. harzianum*** **(BR94)**		***T. koningiopsis* (BR96)**		***End. endophytica*** **(BR98)**	***Pen.oxalicum*** **(BR102)**
*C.* *truncatum*	√	√		√		√		√	√
*C. scovellei*	√	√		√		√		√	√
*F. solani*	√	√		√		√		√	√
*F. oxysporum*	√	√		√		√		√	√
*F. proliferatum*	√	√		√		√		√	√
*F. fujikuroi*	√	√		√		√		√	√
*L. theobromae*	√	√		√		√		√	√
*P. mangiferae*	√	√		√		√		√	√
*L. pseudotheobromae*	√	√		√		√		√	√
*D. pascoei*	√	√		√		√		√	√

**Table 3:** Molecular identification of endophytic fungi isolated from *C. castaneus* spines.

	Genbank Accession Number						
Isolates	ITS	GAPDH	β-Tubulin	TEF-1α	ACT	LSU	% Similarity
*Colletotrichum* spp.							
*C. siamense* BP4	MN635697	MT077122	-	-	-	-	99
*C. siamense* BP8	MN635698	MT077123	-	-	-	-	99
*C. siamense* BP14	MN635699	MT077124	-	-	-	-	99
*C. fructicola* BP5	MN635702	MT077113	-	-	-	-	99
*C. fructicola* SM40	MN635702	MT077114	-	-	-	-	99
*C. endophytica* BP9	MN635726	MT077115	-	-	-	-	99
*C. endophytica* BP10	MN635727	MT077116	-	-	-	-	99
*C. endophytica* BP11	MN635728	MT077117	-	-	-	-	99
*C. endophytica* SM31	MN635729	MT077118	-	-	-	-	99
*C. endophytica* SM33	MN635730	MT077119	-	-	-	-	99–100
*C. endophytica* SM43	MN635731	MT077120	-	-	-	-	99
*C. endophytica* SM44	MN635732	MT077121	-	-	-	-	99
*C. horii* BP3	MN635649	MT077107	-	-	-	-	99
*C. horii* BP7	MN635650	MT077108	-	-	-	-	99
*C. horii* BP12	MN635651	MT077109	-	-	-	-	99
*C. horii* BP13	MN635652	MT077110	-	-	-	-	99
*C. cliviae* SM25	MN652631	MT077111	-	-	-	-	99
*C. cliviae* SM26	MN652632	MT077112	-	-	-	-	99
*C. boninense* SM21	MN635733	MT077106	-	-	-	-	99
*Diaporthe* spp.							
*D. arengae* SM28	MN651480	-	MT077062	MT077093	-	-	98–99
*D. arengae* SM41	MN651481	-	MT077064	MT077095	-	-	98–99
*D. arengae* SM35	MN651483	-	MT077068	MT077099	-	-	98–99
*D. arengae* SM49	MN651487	-	MT077069	MT077089	-	-	98–99
*D. arengae* SM38	MN651484	-	MT077066	MT077097	-	-	98–99
*D. arengae* SM39	MN651485	-	MT077067	MT077098	-	-	98–99
*D. arengae* SM45	MN635732	-	MT077065	MT077096	-	-	97–98
*D. arengae* SM29	MN651486	-	MT077063	MT077094	-	-	98–99
*D. arecae* SM30	MN651482	-	MT077061	MT077090	-	-	99
*D. hongkongensis* SM42	MN651488	-	MT077085	MT077103	-	-	97–99
*D.* cf. *heveae* SM36	MN651489	-	MT077080	MT077092	-	-	96–99
*D.* cf. *heveae* BR74	MN636282	-	MT077079	MT077091	-	-	96–99
*D.* cf. *nobilis* BR67	MN651491	-	MT077084	MT077088	-	-	96–98
*Diaporthe* sp.SM46	MN651495	-	MT077083	MT077100	-	-	98–99
*Diaporthe* sp. SM59	MN651496	-	MT077081	MT077101	-	-	95–99
*Diaporthe* sp. BR103	MN651497	-	MT077082	MT077102	-	-	98–99
*D. tectonae* SM62	MN651493	-	MT077086	MT077104	-	-	95–97
*D. tectonae* SM63	MN651494	-	MT077087	MT077105	-	-	95–98
*Phyllosticta* spp.							
*P. capitalensis* SM20	MN635748	-	-	MT118281	-	-	99
*P. capitalensis* SM23	MN635749	-	-	MT118282	-	-	99
*P. capitalensis* SM32	MN635750	-	-	MT118283	-	-	99–100
*P. capitalensis* SM37	MN635751	-	-	MT118284	-	-	99–100
*P. capitalensis* SM48	MN635752	-	-	MT118285	-	-	99
*P. capitalensis* SM53	MN635753	-	-	MT118286	-	-	99
*P. capitalensis* SM58	MN635754	-	-	MT118287	-	-	99
*P. carochlae* SM27	MN652663	-	-	MT118272	-	-	99
*P. carochlae* SM34	MN652664	-	-	MT118269	-	-	95–99
*P. carochlae* SM51	MN652665	-	-	MT118270	-	-	97–99
*P. carochlae* SM52	MN652666	-	-	MT118271	-	-	97–99
*Neopestalatiopsis* spp.							
*N. saprophytica* BP1	MN635619	-	-	MT264943	-	-	99
*N. formicarum* BP2	MN635621	-	-	MT264929	-	-	99
*N. formicarum* BP6	MN635622	-	-	MT264930	-	-	99
*Trichoderma* spp.							
*T. harzianum* BR93	MN636262	-	-	MT264931	-	-	99–100
*T. harzianum* BR94	MN636263	-	-	MT264932	-	-	99
*T. harzianum* BR95	MN636264	-	-	MT264933	-	-	98–99
*T. harzianum* BR93	MN636262	-	-	MT264931	-	-	99–100
*T. koningiospsis* BR96	MN636269	-	-	MT264934	-	-	99
*T. koningiospsis* BR97	MN636270	-	-	MT264935	-	-	99
*T. koningiospsis* BR99	MN636271	-	-	MT264936	-	-	99
*T.koningiospsis* BR100	MN636272	-	-	MT264937	-	-	99
*Xylaria cubensis*							
*X. cubensis* SM22	-	-	MT118273	-	MT077070	-	99
*X. cubensis* BR84	-	-	MT118274	-	MT077071	-	99
*X. cubensis* BR85	-	-	MT118275	-	MT077072	-	99
*X. cubensis* BR88	-	-	MT118276	-	MT077073	-	99
*X. cubensis* BR89	-	-	MT118277	-	MT077074	-	99
*X. cubensis* BR90	-	-	MT118278	-	MT077075	-	99
*X. cubensis* BR101	-	-	MT118279	-	MT077076	-	99
*X. cubensis* BR105	-	-	MT118280	-	MT077077	-	99
*X. cubensis* BR106	-	-	-	-	MT077078	-	95–99
*Pidoplitchkoviella terricola*							
*Pid. terricola* SM17	MN652667	-	-	-	-	MW338725	96
*Pid. terricola* SM18	MN652668	-	-	-	-	MW338726	96
*Pid. terricola* SM19	MN652669	-	-	-	-	MW338727	96
*Pid. terricola* SM24	MN652670	-	-	-	-	MW338728	96
*Pid. terricola* SM57	MN652671	-	-	-	-	MW338729	96
*Pid. terricola* BR79	MN652672	-	-	-	-	MW338730	96
*Helminthosporium* spp.							
*H. endiandrea* SM61	MT279339	-	-	-	-	MW338667	99
*H. endiandrea* SM64	MT279340	-	-	-	-	MW338668	99
*H. livistonae* BR76	MN652658	-	-	-	-	MW338703	93–97
*H. livistonae* BR78	MN652659	-	-	-	-	MW338704	93–98
*H. livistonae* BR80	MN652660	-	-	-	-	MW338705	93–99
*H. livistonae* BR83	MN652673	-	-	-	-	MW338706	93–99
*H. livistonae* BR87	MT279326	-	-	-	-	MW338669	99–100
*Cladosporium halotolerans*							
*Cla. halotolerans* SM50	MN636281	-	-	-	MT264919	-	99
*Cla. halotolerans* BR75	MN636282	-	-	-	MT264920	-	99
*Penicillium* spp.							
*Pen. indicum* SM65	MN635766	-	MT264923	-	-	-	99
*Pen. indicum* BR91	MN635767	-	MT264924	-	-	-	99
*Pen. oxalicum* BR102	MN636265	-	MT264925	-	-	-	99
*Pen. oxalicum* BR104	MN636266	-	MT264926	-	-	-	99
*Pen. oxalicum* BR107	MN636267	-	MT264927	-	-	-	99
*Pen. oxalicum* BR108	MN636268	-	MT264928	-	-	-	99
*Fusarium* spp.							
*F. lateritium* BR66	-	-	MT296784	MT264940	-	-	99–100
*F. decemcellulare* BR72	-	-	MT296782	MT264938	-	-	99
*F. decemcellulare* BR77	-	-	MT296783	MT264939	-	-	99
*F. lateritium* BR82	-	-	MT296785	MT264941	-	-	99
*F. oxysporum* BR86	-	-	MT296786	MT264942	-	-	99
*F. solani* BR92	-	-	MT296787	MT264944	-	-	99
							
*Cyphellophora guyanensis*							
*Cyp. guyanensis* BR71	MN636279	-	MT264921	-	-	-	99–100
*Cyp. guyanensis* BR73	MN636280	-	MT264922	-	-	-	99
*Arthrinium urticae*							
*Art. urticae* SM47	MN636276	-	-	-	-	-	98–99
*Art. urticae* SM55	MN636277	-	-	-	-	-	98–99
*Art. urticae* SM56	MN636278	-	-	-	-	-	99
*Nemania primolutea*							
*Nem.primolutea* BP15	MN652661	-	-	-	-	-	99
*Nem.primolutea* BP16	MN652662	-	-	-	-	-	99
							
*Cuvularia. lunata* SM54	MN637803	-	-	-	-	-	99
*Muyocopron laterale* SM60	MN637806	-	-	-	-	-	96
*Endomelanconiopsis endophytica* BR98	MN637809	-	-	-	-	-	99
*Acrocalymma fici* BR68	MN637807	-	-	-	-	-	96
*Acrocalymma medicaginis* BR81	MN637808	-	-	-	-	-	96
*Acremonium hennebertii* BR70	MN637805	-	-	-	-	-	99
*Bionectria pityrodes* BR69	MN637804	-	-	-	-	-	99

Note: Colletotrichum endophytica is synonymous with Colletotrichum endophyticum.

**Table 4:** Antagonistic activity of endophytic fungi against plant pathogenic fungi in dual culture test.

	Endophytic Fungi and PGI Value								
Plant Pathogenic Fungi	*C. endophytica* (BP9)	*C. siamense* (BP14)	*C. boninense* (SM21)	*X. cubensis* (SM22)	*X. cubensis* (BR90)	*D. arengae* (SM45)	*D. tectonae* (BR62)	*D.* cf. *nobilis* (BR67)	*D.* cf. *heveae*(BR74)
*C.* * truncatum *	33.33 ± 6.03 ^cd^	13.33 ± 5.58 ^ab^	20.46 1.38 ^bc^	0 ± 0.00 ^a^	1.11 ± 1.72 ^a^	15.24 ± 14.53 ^ab^	45.49 ± 4.04 ^d^	19.57 ± 0.70 ^bc^	38.34 ± 2.40 ^d^
*C. scovellei*	19.52 ± 0.56 ^abc^	55.85 ± 3.27 ^cd^	28.10 ± 6.24 ^bcd^	0.57 ± 1.73 ^a^	1.33 ± 2.37 ^a^	57.73 ± 4.05 ^cd^	70.59 ± 3.51 ^f^	30.55 ± 0.15 ^bcd^	60.50 ± 5.47 ^de^
*F. solani*	35.96 ± 2.15 ^de^	31.58 ± 1.66 ^d^	20.18 ± 2.72 ^bc^	13.16 ± 0.66 ^a^	13.16 ± 2.35 ^a^	16.23 ± 1.98 ^ab^	41.23 ± 2.72 ^e^	17.54 ± 2.15 ^ab^	35.53 ± 2.20 ^de^
*F. oxysporum*	28.47 ± 0.69 ^abc^	49.65 ± 1.57 ^ef^	33.33 ± 1.32 ^bc^	26.39 ± 1.70 ^ab^	20.49 ± 2.77 ^a^	60.76 ± 2.05 ^fg^	61.35 ± 1.66 ^g^	34.72 ± 2.85 ^bc^	57.99 ± 4.04 ^ef^
*F. proliferatum*	28.58 ± 4.01 ^bc^	16.10 ± 0.86 ^abc^	4.45 ± 3.01 ^a^	4.80 ± 3.93 ^a^	6.85 ± 3.88 ^ab^	17.11 ± 15.18 ^abc^	40.45 ± 17.79 ^cd^	14.97 ± 8.87 ^abc^	19.50 4.35 ^abc^
*F. fujikuroi*	41.90 ± 2.76 ^cd^	33.64 ± 8.95 ^abc^	28.44 ± 1.64 ^a^	27.83 ± 1.50 ^a^	25.99 ± 3.96 ^a^	48.93 ± 1.80 ^def^	55.35 ± 6.10 ^ef^	40.06 ± 2.76 ^bcd^	45.8 ± 1.53 ^de^
*L. theobromae*	58.20 ± 5.22 ^ef^	40.23 ± 2.50 ^bc^	50.10 ± 1.00 ^bcde^	55.85 ± 11.90 ^def^	57.40 ± 4.55 ^ef^	38.49 ± 2.86 ^b^	0 ± 0.00 ^a^	0 ± 0.00 ^a^	0 ± 0.00 ^a^
*Pes. mangiferae*	27.78 ± 2.33 ^de^	31.48 ± 1.67 ^ef^	22.22 ± 1.99 ^c^	22.59 ± 1.67 ^c^	22.22 ± 1.99 ^c^	27.04 ± 1.67 ^d^	44.07 ± 1.67 ^g^	29.63 ± 2.30 ^def^	33.33 ± 1.99 ^f^
*L. pseudotheobromae*	43.56 ± 1.38 ^cd^	42.89 ± 1.00 ^cd^	40.22 ± 1.00 ^b^	0.00 ± 0.00 ^a^	0.00 ± 0.00 ^a^	43.56 ± 1.38 ^cd^	56.44 ± 1.38 ^g^	44.44 ± 1.38 ^d^	47.78 ± 1.00 ^e^
*D. pascoei*	39.42 ± 31 ^abc^	38.00 ± 4.88 ^abc^	31.30 ± 2.64 ^ab^	27.82 ± 2.40 ^a^	29.56 ± 1.46 ^ab^	32.75 ± 4.35 ^abc^	38.55 ± 2.38 ^abc^	36.23 ± 2.38 ^abc^	39.13 ± 3.65 ^abc^
**Plant Pathogenic Fungi**	**Endophytic Fungi and PGI Value**								
	***N. saprophytica* (BP1)**	***Pen. indicum* (BR91)**	***T. harzianum* (BR94)**		***T. koningiopsis* (BR96)**		***End. endophytica* (BR98)**		***Pen. oxalicum* (BR102)**
*C.* * truncatum *	19.44 ± 2.51 ^bc^	7.22 ± 2.51 ^ab^	89.33 ± 2.99 ^e^		80.05 ± 5.75 ^e^		53.65 ± 10.85 ^d^		1.34 ± 2.33 ^a^
*C. scovellei*	48.46 ± 8.00 ^cd^	3.20 ± 4.66 ^a^	85.80 ± 5.47 ^e^		89.45 ± 2.55 ^e^		45.70 ± 7.39 ^bcd^		8.09 ± 2.13 ^ab^
*F. solani*	35.96 ± 2.15 ^de^	16.67 ± 11.39 ^ab^	62.28 ± 2.15 ^f^		74.56 ± 2.72 ^g^		24.56 ± 2.72 ^c^		25.44 ± 2.15 ^c^
*F. oxysporum*	46.88 ± 1.14 ^de^	30.56 ± 7.65 ^abc^	76.74 ± 4.45 ^h^		76.04 ± 1.74 ^h^		59.03 ± 5.38 ^fg^		37.85 ± 1.57 ^cd^
*F. proliferatum*	30.18 ± 8.98 ^bcd^	7.94 ± 7.11 ^ab^	57.38 ± 17.22 ^e^		51.63 ± 13.52 ^de^		23.52 ± 8.66 ^abc^		11.36 ± 6.34 ^abc^
*F. fujikuroi*	43.43 ± 6.19 ^cd^	32.42 ± 5.37 ^abc^	71.25 ± 1.50 ^g^		59.94 ± 11.16 ^fg^		46.18 ± 1.50 ^de^		30.28 ± 1.64 ^ab^
*L. theobromae*	43.07 ± 2.89 ^abc^	46.83 ± 0.89 ^abcd^	82.86 ± 1.28 ^f^		77.62 ± 6.30 ^f^		63.59 ± 4.83 ^e^		48.85 ± 3.89 ^abcd^
*Pes. mangiferae*	27.41 ± 2.30 ^cd^	7.41 ± 3.04 ^a^	88.89 ± 1.41 ^g^		60.00 ± 1.99 ^f^		32.52 ± 1.89 ^de^		14.07 ± 2.69 ^b^
*L. pseudotheobromae*	52.44 ± 1.09 ^f^	41.56 ± 1.00 ^bc^	73.78 ± 1.09 ^h^		93.56 ± 1.00 ^i^		53.56 ± 1.00 ^f^		44.44 ± 1.09 ^d^
*D. pascoei*	53.04 ± 6.22 ^d^	39.71 ± 1.809 ^bc^	66.96 ± 1.56 ^e^		66.67 ± 9.30 ^e^		44.35 ± 1.10 ^cd^		39.71 ± 1.809 ^bc^

Superscript letters mean of six replicates, value followed by the same letter are not significantly different (*p* < 0.05) according to Tukey’s test.
